# Phase shift in hippocampal circadian rhythm during the latent period of epileptic rats

**DOI:** 10.1186/1471-2202-12-S1-P76

**Published:** 2011-07-18

**Authors:** David A Stanley, Paul R Carney, Mansi B Parekh, Thomas H Mareci, Sachin S Talathi, William L Ditto

**Affiliations:** 1 School of Biological and Health Systems Engineering, Arizona State University, Tempe, Arizona 85287, USA; 2 Division of Pediatric Neurology, Department of Pediatrics, University of Florida, Gainesville, Florida 32610, USA; 3 Department of Neuroscience, University of Florida, Gainesville, Florida 32610, USA; 4 J Crayton Family Department of Biomedical Engineering, University of Florida, Gainesville, Florida 32610, USA; 5 McKnight Brain Institute, University of Florida, Gainesville, Florida 32610, USA; 6 Department of Biochemistry and Molecular Biology, University of Florida, Gainesville, Florida 32610, USA

## 

Since the late 19^th^ century, clinicians have been aware that epileptic seizures are predisposed to occur at specific times of day. Recent experiments by our group in a chronic limbic epilepsy animal model have lead to the hypothesis that epilepsy following brain injury may emerge as a circadian disorder [1,2]. Specifically, we extracted two distinct classes of population spikes (PS) from CA1 local field potential data. Tracking the firing rate activity of these PS revealed: (a) The rate of PS oscillated with near 24-hour period in both sham controls and epileptic rats. (b) During the epileptogenesis latency period, immediately following electrically induced brain injury, a phase shift greater than 90^0^ materialized between the two classes of population spikes. In addition, we observed a gradually evolving imbalance in which the average firing rate of one PS type increased at the expense of the other over a period of weeks, up until the rat’s first spontaneous seizure [1].

Our present goal is to explain the source of the PS phase shift in relation to the circadian perturbations induced by brain injury. We have constructed a biophysically realistic CA1 network model, consisting of pyramidal (Pyr), basket (B), and oriens-lacunosum molecular (O-LM) cells. Based on evidence from the literature, we consider three circadian-like inputs to the network: GABAergic input from the medial septum, peaking at night (C_MS GABA_); glutamtergic input along the Schaffer collaterals, peaking during the day (C_Schaffer_); and melatonin release, peaking at night and acting to attenuate all GABAergic currents (C_Mel GABA_). All three circadian inputs are implemented by modulating simulation parameters with a scaling factor C_{i}_=[1 + *α* * cos(2*π*(*t* – *t*_0_)/24)].

**Figure 1:**
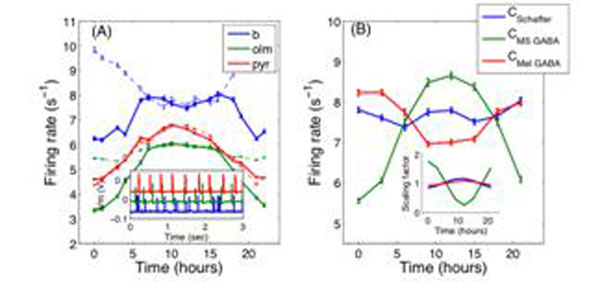
(A) The mean firing rates in the healthy rat (thick) are compared to the injured rat (thin, dotted), showing a 180^0^ phase shift in basket cell firing. Inset shows sample time series from which firing rates were derived. (B) Basket cell firing rate in response to the three individually applied circadian inputs. Removal of C_MS-GABA_ input allows C_Mel-GABA_ signal to take over and produce the 180^0^ shift. Inset: Modulation of circadian scaling factors C_{i}_ used to perturb the system.

The main result of our simulation is presented in Figure 1A. By simulating circadian drive onto the CA1 network of a healthy rat, we show that the firing rates of the three neural populations oscillate in phase (thick lines). For simulation of the circadian perturbations induced by injury, we incorporate our recent high-field brain MRI results. This study, performed in the same animal model, suggested damage to the fimbria-fornix occurred following stimulation. The fimbria-fornix carries the GABAergic septal input. *Removing this input from our model produces an 180^0^ shift in basket cell firing*. Thus, our model provides a possible mechanism (Figure 1B) for the experimentally observed PS phase shift in injured animals, and points to input along the fimbria-fornix as an important source for circadian modulation in the hippocampus.
